# Ligation of External Iliac Artery for Aneurism of the Femoral

**Published:** 1887-03

**Authors:** J. McF. Gaston

**Affiliations:** Atlanta, Ga.; Professor of Surgery


### LIGATION OF EXTERNAL ILIAC ARTERY FOR ANEURISM OF THE FEMORAL.

*Reported for the Southern Medical Record.*

A Clinical Lecture delivered February 2, 1887, at the Southern Medical College, Atlanta, Georgia,

BY J. MCF. GASTON, M. D., PROFESSOR OF SURGERY.

*Gentlemen:* Having witnessed the good result of the first operation upon this patient, by ligation of the femoral artery for the cure of a large popliteal aneurism, I now have the satisfaction of presenting him with favorable prospects, three days after the second operation by ligation of the external iliac, on account of a femoral aneurism some three inches below the pubic bone.

You will recollect that compression was resorted to for a considerable period before the first ligation, and it so turned out that the effects of the continued compression over the pubic bone set up inflammatory action in the adjacent tissues, so that in cutting down upon the line of the artery between the pubic bone and the aneurism, on the 30th ultimo, I encountered adhesions between the coats of the artery and vein, with attachments to the adjoining connective tissues, so that complications were encountered of such a nature as to preclude the completion of the ligation at that point. Not only was the femoral vein opened in the dissection, but it was found impracticable to pass the aneurism needle around with the ligature beneath the artery, on account of its firm agglutination to the subjacent tissues, and I abandoned this undertaking for an operation higher up where the adhesive inflammation had not extended to the vessels or other structures.

While the escape of blood was controlled by pressure upon the femoral below the lesion, I proceeded to divide the tissues upward and ligated the external iliac beneath the border of Poupart's ligament, first with Czerny's silk, moderately tight, and a little lower down with two strands of strong catgut, drawn firmly so as to divide the inner coat of the vessel. You observed, on the occasion, that there was immediate cessation of the pulsation in the aneurism, but that the blood still escaped from the opening in the femoral vein when the pressure was removed, demonstrating the fact that the anastomozing vessels had been so well developed after the first operation two months previously as to restore the blood to the limb. It was, therefore, considered highly important to preserve continuity of the femoral vein, and by the use of sponges with persulphate of iron in the wound, the bleeding ceased.

It will be noted that the ends of both ligatures were left extending from the upper extremity of the incision, as the expectation was to treat the open wound on general antiseptic principles. The carbolized water being used with the sponges in the course of the operation, I applied after the ligation a sponge with undiluted carbolic acid to the entire wound, expecting to get its styptic effect in arresting the venous hemorrhage, but the more potential persulphate of iron was requisite, with compression, to stop the flow of blood. A compress and bandage was left applied, while the watchful supervision of Dr. S. W. Stiles was exercised over the patient subsequently. In the course of three hours he observed that some blood was again escaping from the wound, and renewed the application of the styptic with a fresh sponge, making digital pressure over the tract of the femoral vein just below the aneurismal tumor until my arrival, when a compress was secured over the vessel by a strap around the thigh, drawn only moderately tight. After this there was no further hemorrhage, and the patient manifested no great prostration from the loss of blood, but took a half wineglass of whisky occasionally to relieve the nausea and vomiting following the anæsthetic influence of the A. C. E. mixture used during the somewhat protracted operation, and had a hypodermic of morphia, ¼ grain and atropia, 1-120 grain, applied with benefit. On the subsequent day the patient took chicken soup, and to-day his general condition presents nothing abnormal in temperature or in the pulsation of the wrist, giving evidence of the collateral circulation being established by the warmth of the limb, and natural sensibility in it, with the exception of the little toe and the one next to it, since the first ligation, being insensible to the touch.

We are assured by these phenomena of the great advantage gained by adopting a separate proceeding for each aneurism ; and the indication of impaired peripheral circulation presented by the serous collections about the foot after the ligation of the femoral artery below Scarpa's triangle shows that an operation at the outset above the profunda would most certainly have been followed by deficient nourishment, and loss of vitality in the entire limb.

It is evident now that a thrombus has been formed above the ligation of the iliac, as the impulse of the circulation is no longer perceived in the wound, as it was for some hours after the operation. The dressings have been of simple dry borated cotton with antiseptic gauze, secured by a bandage, since the removal of the sponge at the close of the first twenty-four hours ; and there has been no means of compression used subsequently, so that it is clear that the lesion in the wall of the femoral vein has become closed by adhesive inflammation or the deposition of plastic lymph, thus effectually preventing any return of hemorrhage from this source. The only apprehension of bleeding in the future is from the separation of the ligatures from the artery before a sufficient clot is formed to occlude the tract of the vessel, but the absence of all pulsation for at least two inches above affords ground to expect a favorable result.

*February 10, 1887.*—The observations made since the clinical report bring the history of the case up to the expiration of the 12th day from the operation, and his condition to-day warrants the expectation of his recovery with complete cure of both aneurisms. The double catgut ligature was found entirely disintegrated on the seventh day, and came away in two pieces, thus corroborating the statement heretofore made that catgut ligatures cannot be relied upon for the obliteration of large arteries or for other ligations requiring security for several days. It is evident that this catgut, which had been forwarded to me by my friend Dr. N. Senn, from the house of Schorse & Co., was in a good state of antiseptic preservation. The Czerny's silk ligature, from the same establishment, in Milwaulkee, Wis., continues sound, and is gradually becoming loose by the yielding of the tissues included in the loop, and will doubtless come away very soon. Adhesive strips have been employed to approximate the margins of the incision, since the first week, and the wound is filling up with granulations.

The thoracic symptoms have not given trouble since the third day, and appear to be gradually improving at present, encouraging the hope that there may be no aneurism of the aorta.

*February 15.*—This completes seventeen days since the operation, and the condition of the patient is favorable in all respects. The wound has not suppurated so much as did the closed incision after the first ligation below, and the granulations are shutting in the loop of the ligature, which is not yet detached from the artery; so that slight tension is made upon the loose ends daily, with instruction to attach an elastic if the ligature does not come away in two or three days. Final success is now assured.

---

**The** applications of electricity become more varied every day. Telebarometers, telethermometers, telemanometers and telehydrobarometers have recently been invented, which respectively record, at distant points, air-pressure, heat, steam-pressure and water stages. A California electrician has also invented a process whereby gold, silver and copper can be instantly smelted by a lightning stroke.—*Electrical World.*

